# Sex hormones, intestinal inflammation, and the gut microbiome: Major influencers of the sexual dimorphisms in obesity

**DOI:** 10.3389/fimmu.2022.971048

**Published:** 2022-09-27

**Authors:** Holly Brettle, Vivian Tran, Grant R. Drummond, Ashley E. Franks, Steve Petrovski, Antony Vinh, Maria Jelinic

**Affiliations:** ^1^ Centre for Cardiovascular Biology and Disease Research, Department of Microbiology, Anatomy Physiology and Pharmacology, School of Agriculture, Biomedicine and Environment, La Trobe University, Bundoora, VIC, Australia; ^2^ Department of Microbiology, Anatomy Physiology and Pharmacology, School of Agriculture, Biomedicine and Environment, La Trobe University, Bundoora, VIC, Australia

**Keywords:** leukocytes, obesity, gut microbiota, estrogen (17β-estradiol), testosterone

## Abstract

Obesity is defined as the excessive accumulation of body fat and is associated with an increased risk of developing major health problems such as cardiovascular disease, diabetes and stroke. There are clear sexual dimorphisms in the epidemiology, pathophysiology and sequelae of obesity and its accompanying metabolic disorders, with females often better protected compared to males. This protection has predominantly been attributed to the female sex hormone estrogen and differences in fat distribution. More recently, the sexual dimorphisms of obesity have also been attributed to the differences in the composition and function of the gut microbiota, and the intestinal immune system. This review will comprehensively summarize the pre-clinical and clinical evidence for these sexual dimorphisms and discuss the interplay between sex hormones, intestinal inflammation and the gut microbiome in obesity. Major gaps and limitations of this rapidly growing area of research will also be highlighted in this review.

## Introduction

Obesity is a globally increasing pandemic affecting all ages, ethnicities, sexes, and socio-economic groups. The prevalence of obesity has tripled in the last forty years now affecting ~30% of adults worldwide (1). Obesity is the excessive accumulation of body fat and is associated with an increased risk of developing major health problems such as cardiovascular disease, diabetes and stroke (2). The most used standard in identifying overweight and obesity is a body mass index (BMI; body weight (kg)/height (m) ^2^) > 25 kg/m^2^ classified as overweight and > 30 as obese (3). It is important to note, that while these are the most widely reported BMI cutoffs, they are only relevant to Caucasians. The BMI cutoffs for obesity for other racial and ethnic categories vary to these values (4). For example, the cutoffs for South Asian populations are slightly lower with a BMI > 23 are classified as overweight and > 25 as obese (3). Concomitant metabolic disturbances of obesity include low-grade chronic inflammation, metabolic endotoxemia, hypertension, dyslipidemia, hyperglycemia, and insulin resistance (5). Interestingly, there are clear sexual dimorphisms in the epidemiology and pathophysiology of obesity and its accompanying metabolic disorders. Generally, females are better protected compared to males – this phenomenon will be discussed in much more detail throughout this review (6). Protection in females has been attributed to various biological processes, that will be the focus of this review, such as the influence of adipose distribution, sex hormones, sex chromosomes, the gut microbiota and the intestinal immune system (7–10).

## Adipose tissue biology in obesity

**Figure 1:**
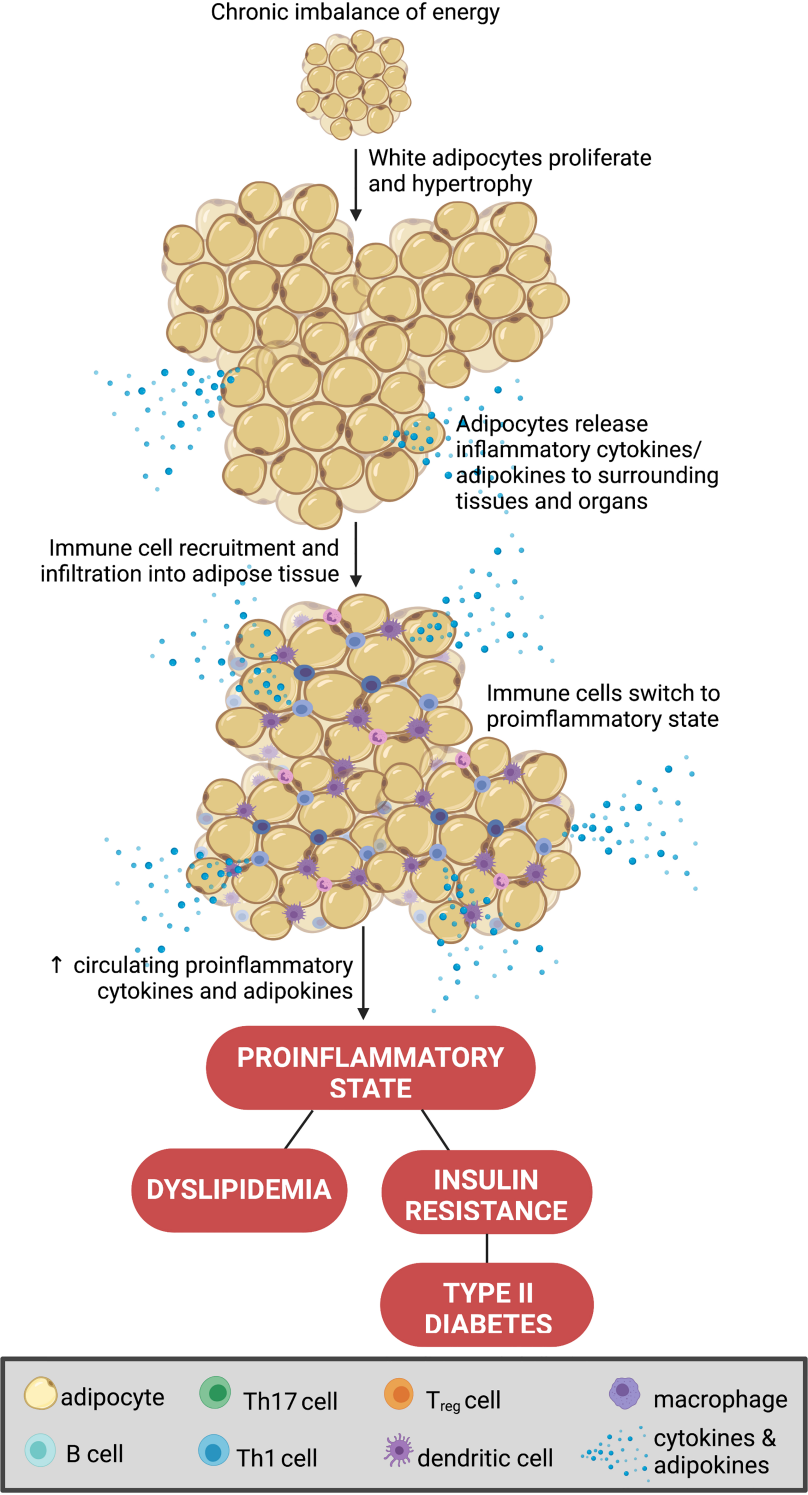
Adipocyte biology in obesity. A chronic imbalance of increased energy intake and or reduced energy expenditure increases adiposity, *via* hypertrophy and proliferation of white adipocytes. This promotes the secretion of pro-inflammatory cytokines (i.e., tumor necrosis factor alpha (TNF-α), interleukin 6 (IL-6), IL-1β, and IL-10) to aid the infiltration of pro-inflammatory immune cells into the adipose tissue and surrounding organs (16). This process promotes dyslipidemia, insulin resistance and hyperglycemia further exacerbating the dysregulation of whole-body energy homeostasis. Created with BioRender.com.

Obesity is instigated by a chronic imbalance of increased energy intake and/or reduced energy expenditure (1). This increases adiposity, a key driver in the development of obesity and the consequential inflammatory state (11). Adipocytes are the predominant cell type in adipose tissue. However, a variety of other cell types also reside in fat beds including leukocytes, endothelial cells and fibroblasts (12). Adipose is a major source of both inflammatory and hormonal signals, and thus is becoming recognized as an endocrine organ in its own right (12). Adipocytes are traditionally classified as either white or brown (12). White adipocytes are particularly important in the storage of energy, whereas brown adipocytes are primarily involved in thermoregulation (via non-shivering thermogenesis) (12). In obesity, where there is a persistent excess of energy, white adipocytes undergo hypertrophy and proliferate to adapt to the accumulation of triglycerides (13). As a result, white adipocytes promote a chronic inflammatory response by secreting pro-inflammatory cytokines such as tumor necrosis factor alpha (TNF-α), interleukin-6 (IL-6), and interleukin-1 beta (IL-1β) (14). This pro-inflammatory phenotype is further compounded by a reduction in the release of anti-inflammatory molecules by obesogenic adipose (15). Ultimately, these changes aid the infiltration of pro-inflammatory immune cells into the adipose tissue and surrounding organs (16). Unsurprisingly, in obesity, white adipose tissue provokes dyslipidemia, insulin resistance and hyperglycemia further exacerbating the dysregulation of whole-body energy homeostasis (16) (**Figure 1**). Importantly, the changes in adipocyte biology and the subsequent downstream metabolic processes in obesity significantly differ between the sexes and therefore, serve as a major source for the sexual dimorphism of obesity (17).

## Sexual dimorphisms in adipose tissue distribution, sex hormones and sex chromosomes

Historically, females have been grossly underrepresented in clinical trials and pre-clinical research. Part of this sex bias in research is the result of an early misconception that men and women are the same. We now know that men and women are unique on a cellular level, and in the setting of obesity there are major sexual dimorphisms. Obesity is slightly more common in females. However, compared to males, females are protected from many of the metabolic disturbances and sequalae that are associated with disease progression in obesity (18, 19). These sexual dimorphisms are also reflected in experimental animal models of diet-induced obesity (19). Male rodents experience an earlier onset and greater degree of obesity, as well as more prevalent concomitant risk factors compared to their female counterparts (such as hyperglycemia, hyperinsulinemia and hypertension) (20, 21). Interestingly, older female animals, or those which model a post-menopause stage (i.e., ovariectomized) are less protected than young females with intact ovaries (22). This correlates with human epidemiology of obesity, whereby men and post-menopausal women are at the greatest risk of developing complications of obesity (23). Collectively, this supports the notion that sex hormones in pre-menopausal women are protective in the setting of obesity. Indeed, sex hormones, such as estrogen, testosterone and androgens are related to the regulation of energy metabolism, food intake and body weight in humans (22, 24). Estrogen is of particular importance and well-established to be protective against cardiometabolic disorders such as obesity, hypertension, and diabetes (25).

**Figure 2:**
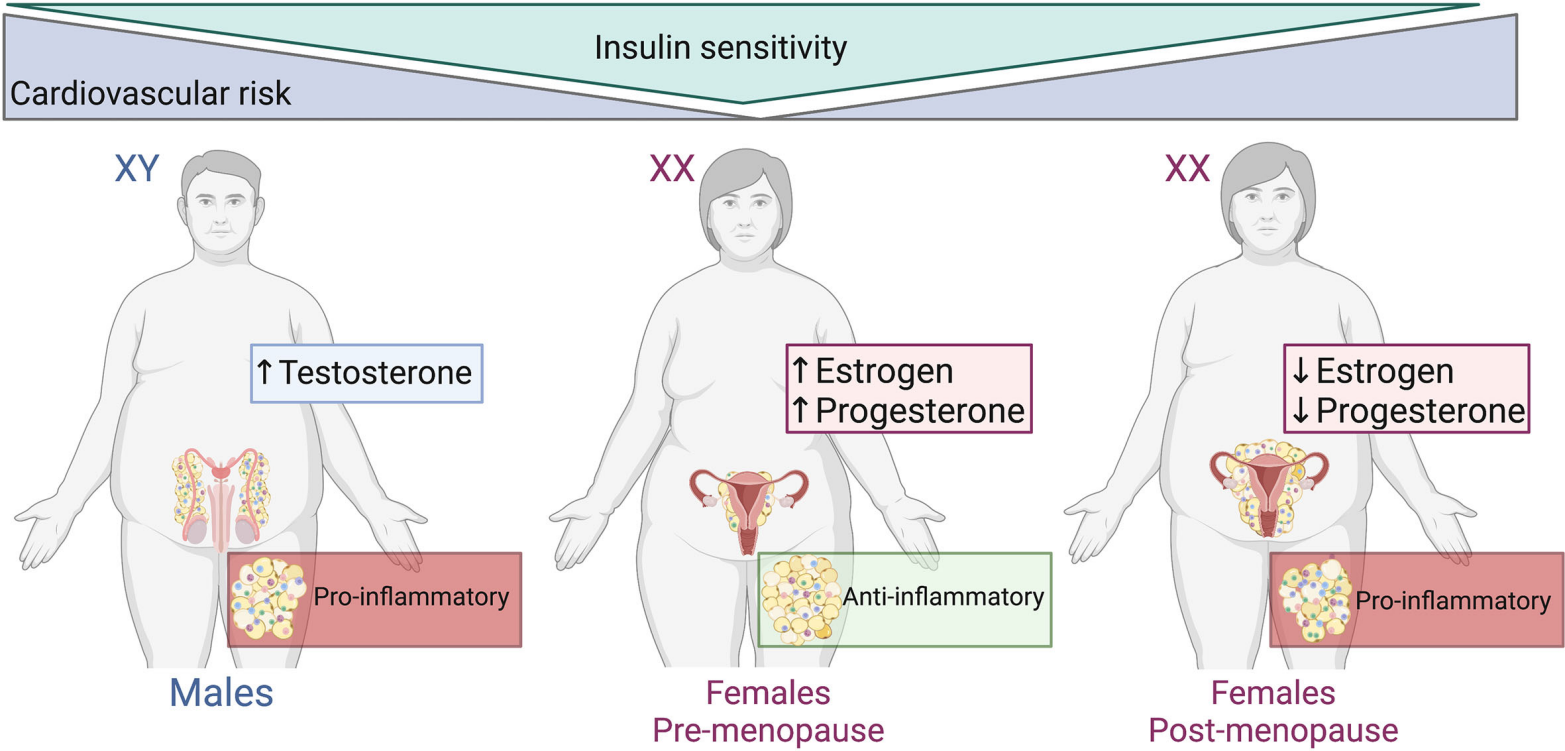
Adipose tissue distribution, sex hormones and metabolic disturbances of obesity. Males and post-menopausal females have increased cardiovascular risk, abdominal/visceral obesity and reduced insulin subcutaneous adipose distribution compared to pre-menopausal females. The adipose tissue within males and post-menopausal females is more pro-inflammatory than that of pre-menopausal females. Created with BioRender.com.

The correlation between adipose tissue distribution, sex hormones and the concomitant metabolic disturbances of obesity are well defined (**Figure 2**), and visceral adiposity is a known driver in the progression of disease in obesity (26). The distribution of adipose tissue throughout the body differs between men and women (27–29). Women have a greater degree of subcutaneous fat (‘gynoid’ pattern), primarily in the gluteofemoral region. Whereas adipose tissue in men is predominantly seen in the abdominal area (‘android’ pattern) as visceral fat (30, 31). The sexual bias of these effects has been reported in both rodent models of obesity and in a clinical setting. Male mice on a high fat diet are at a higher risk of developing a pro-inflammatory profile (visceral inflammation, glucose intolerance, insulin resistance and hyperinsulinemia) when compared to their female counterparts (32, 33). Increased visceral adiposity in men exacerbates the secretion of pro-inflammatory molecules into systemic circulation which produces a knock-on effect whereby the risk of cardiovascular events is markedly increased. This was observed in the European Health Examination Survey in Luxembourg (34). Interestingly, the Netherlands Epidemiology of Obesity Study reported that visceral adipose tissue distribution was more strongly associated with cardiometabolic risk factors in obese females than in obese males (35). The differences observed in these two studies may be due to the Netherlands study including only obese participants, whereas in the Luxembourg study the BMI of participants ranged from <20 to >35 kg/m^2^.

Pre- and post-menopausal studies in women emphasize the role of estrogen in the distribution of adipose tissue by which intra-abdominal visceral fat is increased in post-menopausal women (25, 36–38). With this shift in fat distribution, post-menopausal women undergo metabolic alterations. Lipoprotein lipase activity increases and lipolysis decreases with the fall of estrogen and increased androgenicity is induced during the transition to menopause (36). Ovariectomy in rodents is commonly used as a model of estrogen depletion that occurs in humans. White adipose inflammation is increased and comparable to a male-like phenotype of inflammatory gene expression in ovariectomized mice (39). Despite this pro-inflammatory profile, there were no differences in adipocyte size and total adiposity between ovariectomized and sham mice. This suggests that ovarian hormones are not important in the expansion or apoptosis of adipocytes (39).

In addition to the detrimental effects of visceral adipose, studies also report striking protective effects of gluteofemoral subcutaneous adipose tissue (40). Specifically, increased gluteofemoral mass is associated with lower arterial calcification, arterial stiffness, improved blood lipid levels and atherosclerotic protection (41). While the precise protective mechanisms remain unclear, gluteofemoral adipose has an active role in fatty acid uptake and release by ‘trapping’ excessive fatty acids, preventing lipid accumulation and lipotoxicity (41–43). Lipolysis relative to energy expenditure is therefore higher in women. Other studies link the protective effects of gluteofemoral adipose with the secretion of anti-inflammatory molecules such as adipokines (41).

Sex chromosomes are another crucial contributing factor to the sexual dimorphisms of adipose tissue distribution and the subsequent metabolic complications of obesity. Female gonads typically occur in individuals with XX chromosomes and male gonads in those with XY chromosomes (44). In a unique mouse model, gonadectomized male and female mice carrying XX chromosome complements developed worse obesity disease outcomes than gonadectomized mice carrying the XY chromosome complements (i.e. increased adiposity, increased satiety, and elevated lipid and insulin levels) (45). Gonadectomized mice carrying XO and XXY chromosome complements revealed that the differences between the XX and XY mice due to the additional X chromosome (or “X chromosome dosage”) rather than the lack of a Y chromosome. Indeed, several genes that escape X chromosome inactivation are highly expressed in adipose and liver tissues – both of which are key regulators of metabolism. Thus, the X chromosome may be an important factor in addition to gonads/sex hormones that causes sex differences in obesity and metabolism (45).

Human sex chromosome anomalies also exist such as Klinefelter syndrome (XXY) and Turner syndrome (XO) (46). In Klinefelter syndrome, the most common sex chromosome disorder in men, patients typically present with hypergonadotropic hypogonadism and infertility, with a 5-fold higher incidence in metabolic syndrome, stemming from hypogonadism and low testosterone levels, affecting adiposity and different metabolic traits (47). Turner syndrome patients (the most common sex disorder observed in females, whereby one of the X chromosomes are partially or completely missing) have dramatically reduced gonadal hormone levels. These patients also lack protection against abdominal obesity and have a 4-fold increase in risk for type 2 diabetes (48). Notably, the presence of XX and XY chromosomes influence the developmental path between sexes and gonadal hormones. This ultimately affects the gene expression that may underpin the differences in obesity and metabolism observed between males and females. Although largely attributed to sex hormones and sex chromosomes, the sexual dimorphism of obesity has also been partially credited to sex differences in the microflora residing in our intestines.

## The gut microbiota: A key player in health and disease

The gut microbiota is made up of trillions of complex and dynamic microorganisms living within the intestines and working symbiotically with their host for essential metabolic functions (49). Dietary carbohydrates are fermented by the gut microbiota generating short chain fatty acids (SCFA) as by-products, primarily acetate, butyrate and propionate (50). A higher abundance of SCFA, particularly butyrate, is associated with reduced intestinal inflammation and offers protection against the development of insulin resistance and obesity (51, 52). Additionally, there are certain beneficial, anti-inflammatory bacterial species that respond well to fiber rich diets such as *Akkermansia muciniphila, Bifidobacterium* spp.*, Prevotella* spp., and *Veillonella* spp. forming a favored environment in terms of functionality and immunity (53). Other by-products of the gut microbiota include energy metabolites including pyruvic, citric, fumaric and malic acid (54, 55). These organic acids aid in digestion, immunity, and specifically in preventing the growth of pathogenic bacteria and thus, offer further protection for their host (56, 57).

In addition to aiding in the digestion of foods to produce favorable by-products, the gut microbiota also has an important role in stimulating and regulating hormone production (58). Previous studies show significant correlations between sex steroid levels (i.e., estrogen, progesterone, and testosterone) and gut microbiota composition (7, 59–61). These studies of the interactions between sex hormones and the gut microbiota revealed sexual dimorphisms in the composition of the gut microbiota which will be discussed later. Another crucial function of the gut microbiota is the maintenance of the intestinal immune system response and its tolerance to the bacterial community (62). Due to their close proximity, it is essential that the gut microbiota and intestinal immune system tolerate one another (62). The interaction between the immune system and gut microbiota is a recognized key player in the development of cardiometabolic diseases and will be discussed in detail later in this review. The next section of the review will focus on the role of the gut microbiota in regulating metabolic functions, particularly in the context of diseases such as obesity and other cardiometabolic diseases (63).

The gut microbiota clearly influences the health of its host and various disease states are associated with “dysbiosis” of the gut microbiota (i.e. an altered composition or functionality). However, dysbiosis is often disease-specific and not consistent between different studies. This is likely due to environmental factors such as diet, lifestyle and drugs being major determinants of gut microbiome composition. Consequently, the gut microbiome is highly individualized which makes it difficult to define what constitutes a healthy microbiome (64). Thus, both clinical and experimental studies should be replicated in independent locations to maximize reproducibility and translatability of findings (65). Moreover, it is largely unclear if gut dysbiosis is a cause or the consequence of disease, highlighting the need for further studies defining the molecular mechanisms by which altered microbiomes cause disease. A recent study built a machine learning model that included both human variables and gut microbiota to try to infer gut microbiota and disease associations more accurately (66).

Despite the striking variations in findings between studies, one of the most consistent findings of intestinal dysbiosis in the setting of disease is the loss of microbiota diversity (67, 68). A highly diverse microbiota is thought to be crucial to good gut health as it is more resilient against pathogens, has a greater functionally complex community and builds a stronger and more stable immune system (69–71). Therefore, reduced gut microbiome diversity is most likely detrimental in disease due to a subsequent loss of microbial community function. Many studies have highlighted that microbial community composition is less important than microbial community function. Therefore, increased microbial diversity can be both beneficial or detrimental, more context is often required for accurate interpretation. For example, germ-free mice lacking a microbiome (and thus lack microbiome diversity), but are protected diet-induced obesity, compared to mice with a gut microbiota (72). Ultimately, making conclusions based on microbiota diversity alone has limited value, and should be avoided.

## Major shifts in gut microbiota in obesity

Dysbiosis is a particularly common consequence of a poor diet – a common factor in obesity (73). Diets have a marked influence on the gut microbiota, for example, diets low in fiber and rich in bad fats can modify the bacterial population in as little as 24 hours (74, 75). Diet-induced obesity in animal models is often used to mimic metabolic disturbances and the concomitant gut dysbiosis seen in humans (76, 77). Typically, obesogenic diets include high fat and/or high sugar contents with variations in the types of fat and sugar as well as differences in the duration of diet regimes (77, 78). Importantly, the gut microbiome also influences the concomitant metabolic disturbances of obesity. Oral antibiotic treatment (ampicillin) improves glucose tolerance in high fat diet-fed obese mice. These ‘protective’ effects of antibiotics in obesity are only effective in early life, suggesting that the plasticity of the gut microbiome reduces with age (79, 80).

Gut microbiota dysbiosis describes the imbalance of microorganisms within the gut resulting in metabolic disturbances in the body and contributing to the development of obesity (81, 82). Overall, dysbiosis can be identified by the loss of beneficial bacteria, the increased abundance of harmful bacteria and a loss of compositional and functional diversity (83). Notably, an emphasis has been placed on the status of the Firmicutes: Bacteroidetes ratio, two dominant phyla in the gut microbiota, and how these phyla alter with disease (84). Many studies conclude that disease states such as obesity are associated with an increase in the abundance of the Firmicutes phyla and a decrease in the abundance of the Bacteroidetes phyla (85–87). Moreover, this phenomenon has proven to be reversible with weight loss (88). While the majority of studies report increased Firmicutes : Bacteroidetes ratios in obesity, it is important to highlight that this is not always the case, and contrasting findings have become more common in recent years. For example, in a recent small cohort study of Beijing volunteers the ratio of Firmicutes/Bacteroidetes decreased significantly in people with obesity (89). Larger studies have also reported similar findings (90). Unfortunately, the Firmicutes: Bacteroidetes ratio is not a robust marker of obesity-related microbiome dysbiosis and many of the studies interpreting changes to the Firmicutes: Bacteroidetes ratio are drastically underpowered (91).

A more accurate approach may be to detect obesity-related changes to the genus, family and species levels within the gut microbiome (54). Beneficial bacteria such as *Akkermansia muciniphila* and members of the *Bifidobacterium* genus have a negative correlation in the development of obesity (92, 93). The beneficial effects of *A. muciniphila* on the intestinal epithelial barrier have long been reported, as it a highly effective mucin-degrading bacterium, with the ability to use various enzyme combinations to hydrolyze up to 85% of mucin structures within the gut (94). A reduction in *A. muciniphila* is associated with increased intestinal permeability or a “leaky gut” – a hallmark of gut dysbiosis in obesity (95). Intestinal permeability allows leakage of water, proteins and other endotoxic molecules such as lipopolysaccharide (LPS) into systemic circulation with the ability to reach other organs and tissues (96). High circulating levels of LPS, termed metabolic endotoxemia, promotes further inflammation, weight gain and diabetes in experimental animals and humans (97). Recent studies have explored the possibility of using *A. muciniphila-associated therapies* as a next-generation treatment for obesity (98). Opposingly, harmful bacteria such as the those from the *Desulfovibrio*, *Fusobacterium* and *Bilophila* genera are positively correlated with obesity (92, 93, 99).

## The metabolic and hormonal consequences of gut dysbiosis in obesity

Harmful bacteria within the gut have specified mechanisms that can be destructive to the host. For example, members of the *Desulfovibrio* genus and other sulphate-reducing bacteria induce apoptosis of cells on the intestinal epithelial barrier allowing barrier degradation (100). Additionally, the abundance of gram-negative bacteria increases, with endotoxic lipopolysaccharide (LPS) in their outer membrane (101). LPS then gains access into systemic circulation due to the increased permeability of the epithelial barrier (102). The combination of an increase in harmful bacteria, the decrease in beneficial bacteria, and an increased concentration of pro-inflammatory cytokines within the intestines causes degradation of tight junction proteins between cells allowing LPS and other molecules into underlying tissues and thus, increasing intestinal inflammation (100, 103, 104). Some studies have explored the therapeutic potential of targeting this increase in harmful bacteria in obesity. Alteration of the gut microbiota *via* antibiotics in mice with diet-induced obesity inhibits weight gain, increases lipid oxidation, thermogenesis, and adiponectin gene expression in epididymal adipose tissue. Increases in these molecular pathways likely inhibit fat synthesis and promote a “leaner” phenotype (105). Advances in metagenomics and metabolomics revealed new associations between microbial-derived metabolites (i.e. LPS, short chain fatty acids (SCFAs), ethanol, trimethylamine (TMA), and bile acids) and obesity.

Bile acids are a class of amphipathic steroids synthesized in the liver from cholesterol and metabolized by the gut microbiota. Bile acids facilitate intestinal fat absorption but also modulate glucose, lipid and energy metabolism, intestinal integrity and immunity (106). While there are some discrepancies between studies, circulating bile acid levels are generally positively correlated with obesity. Importantly, microbiome-derived bile acid species have different signaling functions to liver-derived species. There is a growing body of evidence suggesting a link between the microbiome – an important player in bile acid metabolism – and bile acid levels/composition in obesity (106). Fecal microbiota transplantation (FMT; from a single lean donor) in obese, metabolically uncompromised patients had sustained shifts in microbiomes and bile acid levels toward those of the donor (107). Like much of the microbiome research to date, further studies are still needed to establish whether there is causality, as these “beneficial” changes were not associated with weight loss or changes to glucose metabolism.

To date, numerous studies suggest that gut microbiomes influence eating behavior in humans and animals. Appetite-related hormones such leptin (inhibits appetite) and ghrelin (promotes appetite) are produced by peripheral organs, including gut and adipose tissue. Changes to specific microbial compositions have reported effects on these hormones, and *vice versa*. For example, in obese and non-obese humans, higher circulating leptin concentrations are associated with reduced gut microbiome diversity (108). Moreover, *in vivo* and *in vitro* studies showed that the translocation of living gut microbiota to adipose tissues in obese patients with increased intestinal permeability inhibits leptin signaling (109). Alternatively, the gut microbiota may modulate appetite *via* grehlin. In another study, treatment with SCFAs, lactate, or bacterial supernatants to promote gut microbiome health attenuated ghrelin-mediated signaling (110).

Clearly, whilst gut dysbiosis has been consistently reported in obesity, the severity, and subsequent consequences of dysbiosis vary among obese individuals depend on many factors, which likely explains inconsistent findings between studies (111, 112). One such factor that has more recently been recognized to influence the gut microbiota is an individual’s sex.

## Sexual dimorphisms and gut microbiota

The impact of the gut microbiota and its influence on the development of obesity has been well documented. However, one aspect that was overlooked in earlier research is the effect of sex. Many studies have investigated the impact of the gut microbiota by altering variables such as diet, lifestyle, and drugs but it is important to recognize that the gut microbiota is different for males and females prior to any manipulations (113). Sequencing the microbial community of prepubescent male and female mice does not show any separation between sexes indicating that sex differences are influenced by gonadal-derived sex hormones and puberty (59, 114). Typically, in mice, the female gut microbiota more closely resembles that of prepubescent males, or castrated males, rather than age-matched males (59, 115). Furthermore, the diversity differs between sexes, with males having a lower species richness and evenness compared to females of the same age in mice models (116). As well as differences in diversity, both animal and human studies show clear variance in the abundance of specific bacteria being higher in one sex compared to the other (113, 117–119). The distinct differences in the male and female gut microbiota, for both animal and human models, inevitably generate differences in metabolic processes and therefore, differences in dysbiosis and the protection or susceptibility to metabolic diseases including obesity (56, 115, 120).

It is well-established that sex steroid hormones are the major drivers of sexual dimorphisms in males and females however, whether there is the strong interaction between sex hormones and gut microbiota is still unclear (59). An observational study that compared the microbiota of men and women with higher serum hormone levels to those with low hormone levels suggests that sex hormones do indeed influence the gut microbiota (121). Higher levels of hormones were associated with a greater diversity in the gut community compared to those with lower hormone levels in both sexes. Moreover, bacteria such as those from the *Acinetobacter, Ruminococcus* and *Megamonas* genus were significantly associated with testosterone levels in men and *Slackia* and *Butyricimonas* were significantly associated with estradiol levels in women (121). Gut microbial transplants to the opposite sex have also been used to determine the hormonal association (10). In these studies females receiving male donor gut microbiota, not only showed higher levels of gut inflammation (a common sign of obesity and cardiometabolic disease) but also resulted in raised testosterone levels (10, 115).

The gut microbiota has also been shown to directly influence sex hormone levels in animal studies using microbial transplants between germ-free mice and mice of opposite sex (7, 10, 122). Colonizing germ-free mice with gut microbiota increases the levels of circulating androgens and begins the development of immune and protective pathways (7, 59). However, there is also evidence that sex hormones can also influence the gut microbiome. Inoculating germ-free mice with human male donor gut microbiota results in males and females harboring these microorganisms differently (122). In female mice, the gut microbiota significantly differed from the matched males and donor, with a higher bacterial diversity (122). Collectively, these studies indicate that there is likely a two-way communication between systemic sex hormones and the gut microbiome, whereby both factors impact one another.

The interactions between sex hormones and gut microbiota have also been studied in animal models using hormone and gonadectomy treatments (61, 101). Estradiol, the most common form of estrogen, is used in hormone treatments to remedy the loss of ovarian estrogen typically seen in menopausal women (123). High fat diet-fed female mice treated with estradiol are protected from cardiometabolic disease (reduced weight gain, improved glucose tolerance and insulin sensitivity) when compared to untreated high fat diet-fed female mice (61). Moreover, estradiol alters the gut microbiota by slowing the increase in Firmicutes: Bacteroidetes ratio that is usually seen in high fat diet-fed mice (61). Interestingly, sequencing of the gut microbiome of these mice revealed that bacteria from the *S24-7* and *Ruminococcaceae* families, known to generate beneficial SCFA, were in higher abundance in estradiol-treated mice, compared to untreated mice (61). The benefits of estradiol treatment are not limited to just females. Male mice treated with estradiol have a reduced susceptibility to gut epithelial permeability, inflammation and weight gain compared to untreated males (101, 124).

## Sexual dimorphisms of the gut microbiota in obesity

As previously discussed, the female sex is also protected from the development of metabolic disturbances in obesity, and the gut microbiota responds differently to diet based on sex (summarized in **Table 1**). This was demonstrated in overweight and obese adults undergoing either a high protein or low-fat weight loss intervention diet (125). Changes in the gut microbiota occurred not only in diet-specific manner but also differed based on sex (125). Additionally, in animal models of obesity, high fat/high sugar diet-fed mice, demonstrated that females respond slower to the biological adverse effects of the diet as well as differentiating in the composition of the gut microbiota, compared to males (126). The increased Firmicutes: Bacteroidetes ratio typically seen in the development of obesity and metabolic disease is significantly slower in female mice (127). Moreover, differences in the abundance of specific genera are also observed in metabolic syndrome patients. Higher abundances of *Veillonella*, *Methanobrevibacter, Acidaminococcus, Clostridium, Roseburia* and *Faecalibacterium* genera in males, whereas genera such as *Bilophila, Ruminococcus* and *Bacteroides* were greater in females (68, 82). In the male gut microbiota for example, *Veillonella* genera are found in higher abundance in children with type 1 diabetes however, *Roseburia* genera is found to improve metabolic alterations brought on by high fat diets (128, 129). Likewise, for females, *Bilophila* genera aggravates metabolic dysfunction however, *Bacteroides* genera has numerous metabolic benefits on the host (130, 131). These findings suggest that it is not simply the abundance of specific bacteria in the gut microbiota that determine health and disease within the host.

**Table 1:** Summary of human and mouse studies investigating the sexual dimorphisms of intestinal microbiota in obesity.

Model	Age	Physiological effects	Main findings	Ref
4-months of either moderately high-protein or LFD in patients with BMI > 25kg/m^2^	N/A	Weight loss: ↓ BMI, total & visceral fat, BP, total glucose, LDL cholesterol, leptin, and insulin regardless of diet or sex.	Weight loss-related changes to the intestinal microbiota occurred in a sex- and diet-specific manner.	(125)
Men & post-menopausal women, split based on BMI, following either a Mediterranean or low-fat diet.	♂: 61.2±1.3y ♀: 60.3±1.4y	N/A: study did not compare physiological parameters between sexes or groups.	Obesity influenced sex differences in gut microbiota. ♂: ↓ *Bacteroides* abundance with ↑ BMI; ↑ *Methanobrevibacter* abundance *(vs.* ♀) regardless of BMI. ♀: ↔ *Bacteroides* abundance with ↑ BMI; ↑ *Bilophila* abundance *(vs.* ♂) regardless of BMI.	(82)
C57BL/6 mice fed either a NCD or HFD (60% fat) for 20 weeks.	8 weeks old	♂: ↑ weight gain for the first 7 weeks on HFD *vs.* ♀. No sex differences following this timepoint.	Sex differences existed in diversity and structure of the gut microbiota at baseline. Gender-specific changes to gut microbiota occurred following HFD.	(113)
C57BL/6 mice fed either a LFLS (10% total fat) or HFHS (45% total fat) for 14 weeks.	8 weeks old	♂: HFHS ↑ weight gain and plasma leptin *vs.* ♀ HFHS mice.	Significant differences in gut microbiota between males and females in both LFLS and HFHS groups. Diet-induced changes to Firmicutes differed between males and females for certain genera.	(126)
C57BL/6 mice fed either a LFD (10% total fat) or HFD (60% total fat) for 20 weeks.	6 weeks old	HFD increased body weight in males and females however, males developed obesity much earlier than female mice.	♂: HFD ↓ Bacteroidetes, Proteobacteria & Tenericutes; ↑ Firmicutes. LFD ↓ Proteobacteria & Tenericutes; ↑ Bacteroidetes; ↔ Firmicutes. ♀: HFD ↓ Firmicutes & Tenericutes; ↑ “others”; ↔ Bacteroidetes. LFD ↓ Firmicutes & Tenericutes; ↑ Bacteroidetes & “others”.	(127)

BMI, body mass index; BP, blood pressure; HFD, high fat diet; HFHS, high fat, high sugar diet; LDL, low-density lipoprotein; LFD, low fat diet; LFLS, low fat, low sugar diet; N/A, not applicable; NCD, normal control diet; ref, reference.♂, male; ♀, female; ↑, increased; ↓, decreased; ↔, unchanged.

In addition to the sexual dimorphism in response to poor “Western-style” diets, the sex-specific response to beneficial diet supplementations have also been studied (132, 133). Beneficial fiber compounds, including pre- and probiotics, can attenuate the unfavorable effects of the diet by shaping the gut microbiota (93). The addition of prebiotic fibers, such as oligofructose, significantly increases beneficial gut bacteria in healthy and gnotobiotic female, but not male mice, such as *Bacteroides* and *Bifidobacterium* genera and *A. muciniphila* (122, 134). Furthermore, probiotic treatments also adjust the gut microbiota differently for males and females (135). Administration of *Lactobacillus reuteri* increased the abundance of the Bacteroidetes phylum and decreased Firmicutes in healthy female mice, but showed opposite effects in healthy male mice (135). Given the differences at the phylum level, this also incurred significant differences at the genus level. Females had a significantly greater abundance of *Bacteroides, Prevotella* and *Lactobacillus*, but males had a higher abundance of C*lostridium* (135). These findings not only illustrate that the female gut microbiota has a stronger protection against the adversities of poor diet, but also that the male and female gut microbiota respond differently to the effect of beneficial supplements and how they harbor their microbial communities. The explanation for the sexual dimorphisms in gut microbial composition and function, in both healthy and metabolically disturbed subjects, comes full circle and back to differences in sex steroid hormones and inflammatory responses.

**Figure 3:**
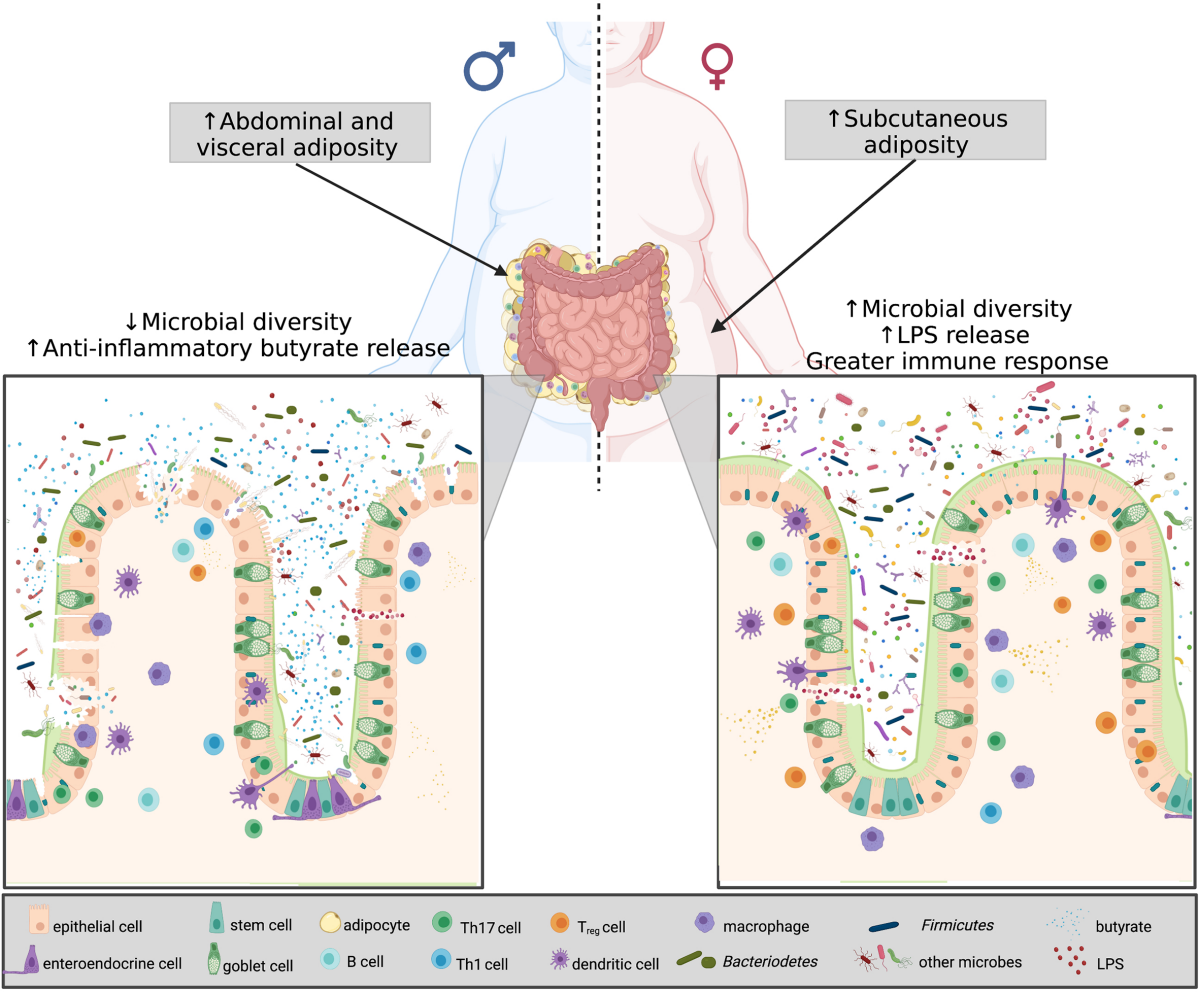
Microbial diversity, sex hormones and chromosomes in obesity. Differences in sex-based characteristics are modulated by a variety of factors. Women have a greater degree of subcutaneous fat, whereas males predominantly accumulate visceral fat. In obesity, the shift in the Firmicutes: Bacteroidetes determines disease severity. Obese males have less species richness, and testosterone was found to be associated with increased Firmicutes, thus more anti-inflammatory butyrate release. Obese females, on the other hand, despite having greater microbial diversity, have an increase estradiol and Bacteroidetes, resulting in greater LPS release, thus eliciting a greater immune response. Created with BioRender.com.

Gonadectomy surgery can be used to eliminate sex steroid hormones and therefore, also be used to study the interactions between sex hormones and the gut microbiota in obesity. In ovariectomized obese female mice, the Firmicutes phyla dominated the gut microbiota community which is commonly seen in obese and high fat diet-fed mice and the sequenced gut community of ovariectomized female mice more closely resembles that of male mice (101, 136). Furthermore, when treating ovariectomized female mice and male mice with estrogen the microbial composition resembles that of non-ovariectomized female mice (101). Similar to ovariectomized mice and the reduction in estrogen, is the changes occurring to the gut microbiota with menopause (137). Studies have shown that the gut microbiota of post-menopausal women reveal higher abundances of Firmicutes compared to both pre-menopausal women and age-matched males (137). These findings reveal that sex and sex hormones, specifically in the presence of obesity, strongly guide the shape of the gut microbiota. In addition to the sexual dimorphism existing within the gut microbiota and obesity, research has revealed that sex-based differences of obesity are also associated with the immune system specifically within the gut (**Figure 3**).

## Sexual dimorphism of intestinal inflammation

Obesity is commonly accompanied with low-grade systemic inflammation which is a key driver of the subsequent comorbidities of obesity due to higher concentrations of endotoxic molecules (i.e., LPS from bacteria) and in circulation increased adiposity increasing cytokines such as TNF-α, IL-1, and IL-6 (138, 139). Many studies in obesity have concentrated on visceral adipose tissue as the driving force of inflammation however, inflammation within the intestinal tract precedes both adipose tissue inflammation and obese characteristics such as weight gain (138). This finding is of particular importance as a significant proportion of the systemic innate and adaptive immune cells within the body (70%) reside within the intestinal tract (140). To our knowledge, the sexual dimorphisms of the intestinal immune system in the setting of obesity has not been researched in a preclinical setting. However, studies have identified sex differences in healthy individuals (141, 142). For example, in the lamina propria layer of the intestines female have higher immune activation and higher CD4^+^ and CD8^+^ T cell counts in compared to males (141).

Another crucial mediator of the sexual dimorphisms in intestinal immunity is the gut microbiota. Due to their close proximity, the interplay between the gut microbiota and intestinal immune system is well-established as shaping and developing one another (143). This is highlighted in studies using germ-free mice, which lack a gut microbiota. The consequence of this is poorly developed intestinal lymphatic tissue (Peyer’s patches) and immune cell populations (144). Moreover, the sex differences of intestinal immunity in autoimmune disease settings are abolished in germ-free mice, suggesting that the sex bias in immunity is driven by sex differences in the microbiome rather than sex hormones (59, 115).

As mentioned previously, females have a stronger intestinal immune response compared to males and this influence of the gut microbiota on this must also be considered (145). Therefore, the sexual dimorphisms of the gut microbiota and in particular, the difference in biomarkers of obesity such as the Firmicutes: Bacteroidetes phyla and taxa abundance difference likely drive the discrepancies in the intestinal immune system of males and females (127). For example, the Firmicutes phyla are the predominant producers of butyrate, a known anti-inflammatory molecular metabolite (146). Therefore, the increased Firmicutes abundance typical of obese males (compared to obese females), elevates butyrate production, which could suppress the intestinal immune response in males. Alternatively, the Bacteroidetes phyla, generally seen in higher abundance in obese females compared to obese males, are gram-negative bacteria (147). Gram-negative bacteria contain LPS in their outer membrane thus, an increased abundance of these taxa, and subsequent increased circulating LPS, correlates with a stronger intestinal immune response (147).

Although a stronger immune response is associated with an increased inflammatory profile, this may be beneficial in the context of obesity and intestinal inflammation. For example, females are superior in eliminating pathogenic and opportunistic bacteria (possibly obesity-related bacteria) present in the gut, which might be a by-product of their enhanced immune response. The enhanced immune response in females may very well be the factor that protects or delays the development of obesity-related metabolic disturbances in females (148). In the opposing manner, the intestinal immune response is relatively smaller in males, thus allowing the manifestation of deleterious microorganisms and thus, possibly exacerbating the disease development of obesity.

## Conclusion

**Figure 4:**
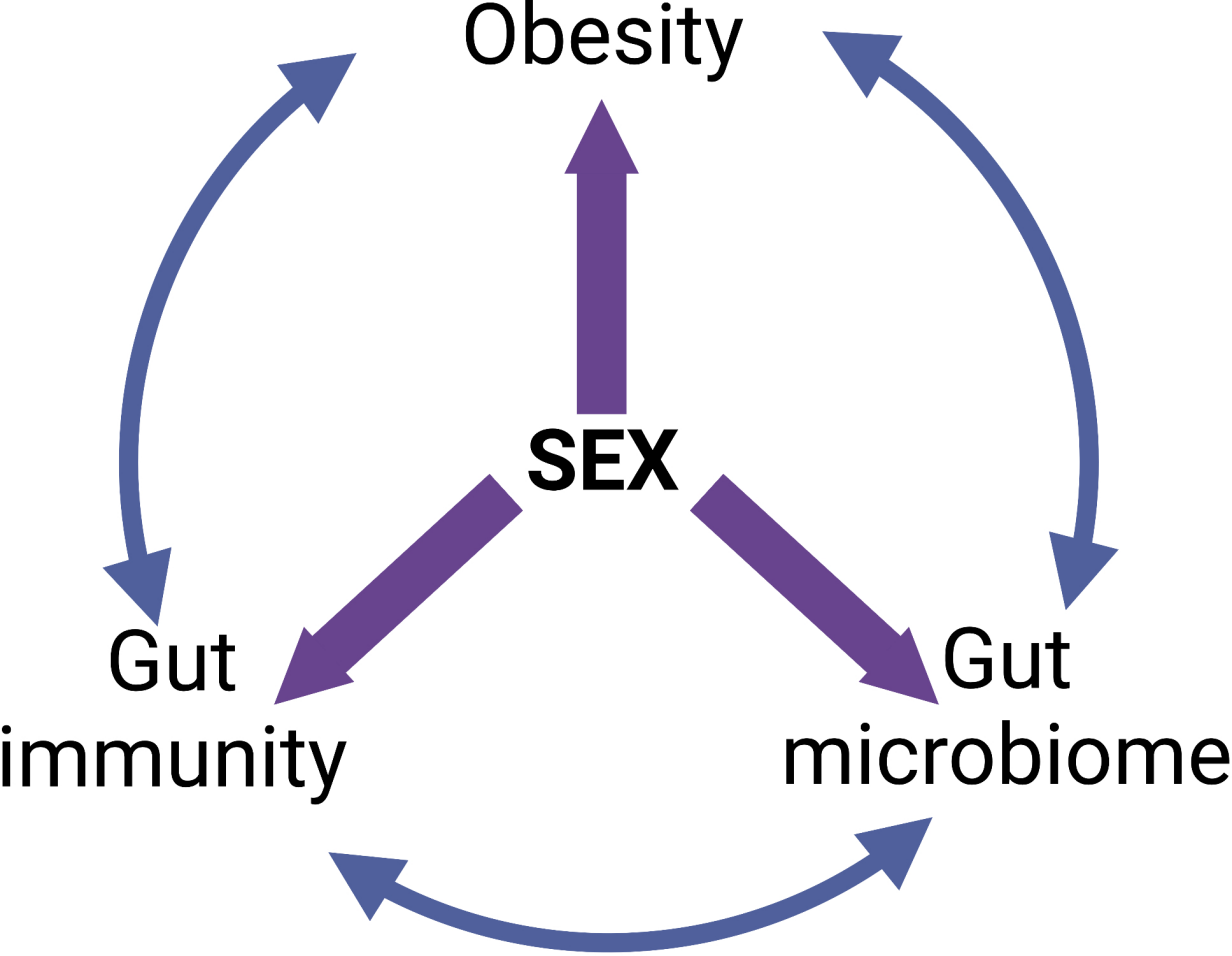
The “give-and-take” relationships between obesity, intestinal immunity, gut microbiome and sex. Created with BioRender.com.

The sexual dimorphisms in the epidemiology and pathophysiology of obesity put males and post-menopausal women at the greatest risk of metabolic disturbances and end-organ damage. Although several factors such as sex hormones, sex chromosomes and fat distribution serve as a basis for these sexual dimorphisms, they can also be attributed to differences in the composition and function of the gut microbiota and the intestinal immune response. Both the gut microbiota and immune system are well-documented influencers of the development of obesity however, the important role that sex plays in this relationship is often overlooked. The “give-and-take” relationship each of these three factors have on one another is an important consideration for future studies (**Figure 4**). Moreover, the vast majority of studies to date are purely associative. More studies that assess causality are needed to unequivocally identify which harmful gut bacteria and or specific gut microbiome imbalances cause obesity. Importantly, it is crucial that these causal studies firstly consider the sex differences in the gut microbiota prior to commencing the study; and secondly, assess the role that sex plays throughout the treatment that will influence the study outcomes. In addition to this, the sex differences in the intestinal immune response in obesity must also be considered in future studies. Very few studies examine both the microbiome and intestinal immune response. Finally, due to the sexual dimorphisms that exist in both the gut microbiota and intestinal immune response, it is crucial that females – both pre- and post-menopausal – are represented in research studies to the same extent as males for findings and future treatments to be valid in both sexes.

## Author contributions

HB wrote the first draft of the manuscript. VT, AV and MJ wrote sections of the manuscript. HB, VT and MJ created the figures. All authors contributed to manuscript revision, read, and approved the submitted version.

## Funding

HB and VT were funded by Australian Research Training Scholarships. MJ was funded by a joint NHMRC and National Heart Foundation Early Career Fellowship (GNT1146314 and 101943, respectively).

## Conflict of interest

The authors declare that the research was conducted in the absence of any commercial or financial relationships that could be construed as a potential conflict of interest.

## Publisher’s note

All claims expressed in this article are solely those of the authors and do not necessarily represent those of their affiliated organizations, or those of the publisher, the editors and the reviewers. Any product that may be evaluated in this article, or claim that may be made by its manufacturer, is not guaranteed or endorsed by the publisher.
